# An Unprecedented Challenge: The North Italian Gastroenterologist Response to COVID-19

**DOI:** 10.3390/jcm11010109

**Published:** 2021-12-25

**Authors:** Gian Eugenio Tontini, Giovanni Aldinio, Nicoletta Nandi, Alessandro Rimondi, Dario Consonni, Massimo Iavarone, Paolo Cantù, Angelo Sangiovanni, Pietro Lampertico, Maurizio Vecchi

**Affiliations:** 1 Gastroenterology and Endoscopy Unit, Fondazione IRCCS Ca’ Granda Ospedale Maggiore Policlinico, 20100 Milan, Italy; giovanni.aldinio@unimi.it (G.A.); nicoletta.nandi@unimi.it (N.N.); alessandro.rimondi@unimi.it (A.R.); maurizio.vecchi@unimi.it (M.V.); 2 Department of Pathophysiology and Organ Transplantation, University of Milan, 20100 Milan, Italy; paolo.cantu@policlinico.mi.it (P.C.); pietro.lampertico@unimi.it (P.L.); 3 Epidemiology Unit, Fondazione IRCCS Ca’ Granda Ospedale Maggiore Policlinico, 20100 Milan, Italy; dario.consonni@policlinico.mi.it; 4 Gastroenterology and Hepatology Unit, Fondazione IRCCS Ca’ Granda Ospedale Maggiore Policlinico, 20100 Milan, Italy; massimo.iavarone@policlinico.mi.it (M.I.); angelo.sangiovanni@policlinico.mi.it (A.S.)

**Keywords:** COVID-19, SARS-CoV-2, gastroenterology, hepatology, endoscopy, delivery of healthcare

## Abstract

Background: COVID-19 pandemic has profoundly changed the activities and daily clinical scenarios, subverting organizational requirements of our Gastroenterology Units. AIM: to evaluate the clinical needs and outcomes of the gastroenterological ward metamorphosis during the COVID-19 outbreaks in a high incidence scenario. Methods: we compared the pertinence of gastroenterological hospitalization, modality of access, mortality rate, days of hospitalization, diagnostic and interventional procedures, age, Charlson comorbidity index, and frequency of SARS-CoV-2 infections in patients and healthcare personnel across the first and the second COVID-19 outbreaks in a COVID-free gastroenterological ward in the metropolitan area of Milan, that was hit first and hardest during the first COVID-19 outbreak since March 2020. Results: pertinence of gastroenterological hospitalization decreased both during the first and, to a lesser degree, the second SARS-CoV2 waves as compared to the pre-COVID era (43.6, 85.4, and 96.2%, respectively), as occurred to the admissions from domicile, while age, comorbidities, length of stay and mortality increased. Endoscopic and interventional radiology procedures declined only during the first wave. Hospitalized patients resulted positive to a SARS-CoV-2 nasopharyngeal swab in 10.2% of cases during the first COVID-19 outbreak after a median of 7 days since admission (range 1–15 days) and only 1 out of 318 patients during the second wave (6 days after admission). During the first wave, 19.5% of healthcare workers tested positive for SARS-CoV-2. Conclusions: a sudden metamorphosis of the gastroenterological ward was observed during the first COVID-19 outbreak with a marked reduction in the gastroenterological pertinence at the admission, together with an increase in patients’ age and multidisciplinary complexity, hospital stays, and mortality, and a substantial risk of developing a SARS-CoV-2 test positivity. This lesson paved the way for the efficiency of hospital safety protocols and admission management, which contributed to the improved outcomes recorded during the second COVID-19 wave.

## 1. Introduction

COVID-19 pandemic has profoundly changed the activities and daily clinical scenarios, subverting essential clinical and organizational requirements of all hospital units. As of today, there are only a few studies describing the features and consequences of COVID-related re-organization of Gastroenterology departments [1,2], but there are no experiences describing both the changes that occurred in these settings, as well as the consequent adjustments applied and their impact on hospitalized patients and healthcare personnel. 

During the firsts SARS-CoV2 outbreak, the Internal Medicine Units and other specialized units were suddenly converted into COVID Units, while other specialized Units, such as Gastroenterology Units and the Nephrology Units, became COVID-free wards with dedicated safety protocols to guarantee adequate inpatients assistance for a broad range of clinical presentations.

We evaluated the clinical needs and outcomes of a COVID-free gastroenterological ward of a hospital in the metropolitan area of Milan that was hit first and hardest during the first COVID-19 outbreak since March 2020 [3], before the availability of the first SARS-CoV-2 vaccinations (27 December 2020 for healthcare workers and only afterward for the general population). 

We hypothesized a sudden metamorphosis of the gastroenterological clinical practice towards a situation that resembles an Internal Medicine and a Geriatric Unit in the pre-COVID era, with a marked reduction in the gastroenterological pertinence and elective admissions, together with an increase in hospital stays and mortality. 

## 2. Material and Methods

Hospital charts related to hospitalized patients in January and February 2020 were used as a model for the pre-COVID era. Patients admitted between March and April, in September and from October to December 2020 represent, respectively, the first wave, the transition period, and the second wave of COVID-19 pandemic according to the regional epidemiological trends in the general population. 

First, we assessed and compared the pertinence of hospitalization within the gastroenterological ward during different periods across the first and the second SARS-CoV2 outbreaks. 

Secondly, we evaluated modality of access, mortality, days of hospitalization, gastroenterological diagnostic and operative procedures, age, Charlson comorbidity index (CCI), and frequency of SARS-CoV-2 infections in patients and healthcare personnel within the gastroenterological ward based on a molecular test performed on a nasopharyngeal swab. This monocentric retrospective cohort study was performed in the gastroenterological ward of a tertiary referral university hospital located in the city center of Milan (Italy), which encompasses a Gastroenterology and Endoscopy Unit and a Gastroenterology and Hepatology Unit.

Safety measures adopted in the gastroenterological ward to face the COVID-19 outbreak and protect both patients and healthcare workers were reported in detail in the Supplementary Methods.

The inclusion criteria were adult age (>18 years old), being hospitalized in the Gastroenterology Units from 1 January 1 2020 to 30 April 2020 and from 1 September 2020 to 31 December 2020.

In the absence of a validated definition in literature, the gastroenterological pertinence of the diagnoses was defined a priori as “any primary or secondary condition that determines a clinically significant dysfunction of the gastrointestinal system.”

For each patient, the following data were collected: sex, age, entry and exit dates in the Unit; modality of access (collected as 2 categories: from domicile and others including Emergency Department, other Units, other Hospitals); discharge diagnosis; CCI [4,5], date of the positive nasopharyngeal swab for SARS-CoV-2; endoscopic and other interventional procedures (e.g., trans-arterial chemoembolization, TACE) performed throughout the hospitalization period in our Units. 

In June 2020, healthcare workers received a questionnaire assessing their involvement in the Gastroenterological ward from 1 January 2020 to 30 April 2020 to weigh their potential worker exposure to SARS-CoV2 infection during the first wave (i.e., hours per week with direct involvement within the Gastroenterological ward).

Descriptive data were expressed as counts and percentages for categorical variables, as medians and ranges for continuous variables. The chi-squared test was used to analyze dichotomous variables. Univariate log-binomial regression models were used to calculate prevalence ratios (PR) and 95% confidence intervals (CI) for different periods vs. the pre-COVID period. The Kruskal–Wallis test was applied for the analysis of quantitative variables in the 4 periods. Statistical analyses were performed using Stata 17 (StataCorp., College Station, TX, USA, 2021).

The study was carried out in accordance with the Declaration of Helsinki adopted in 1964, incorporating all later amendments after formal approbation from the local Ethical Committee (Comitato Etico Milano Area 2, 19 May 2020; ID1588). All participants gave informed consent to participate in the study according to the study protocol.

## 3. Results

**Figure 1:**
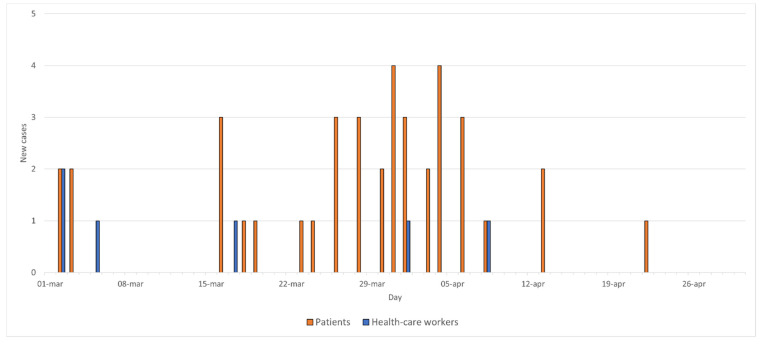
Daily incidence of SARS-CoV-2 during the first wave (SARS-CoV-2 molecular nasopharyngeal swab positive tests).

The total number of recruited patients in the study periods was 699, of which 426 males and 273 females, with a median age of 68 years (range 17 to 98 years) (Table 1). From March to April 2020, 39 out of 381 patients (10.2%) resulted positive to SARS-CoV-2 testing with a molecular nasopharyngeal swab after a median length of stay of 7 days (range 1–200) (Figure 1). From September to December 2020, only 1 out of 318 patients (0.003%) resulted positive to SARS-CoV-2 after 6 days spent in the gastroenterological ward. Notably, most of them (35/40) had at least one molecular nasopharyngeal swab negative for SARS-CoV-2 performed before ward admission (i.e., emergency room or pre-hospital triage), while 4 cases occurred in patients admitted a few days before the adoption of a systematic SARS-CoV-2 pre-hospital triage when the first COVID19 outbreak was already in progress but still largely unexpected. Among the 40 patients who tested positive for SARS-CoV-2, 27 (67.5%) had respiratory symptoms (at least one among cough, dyspnea, and mild respiratory insufficiency) at the time of hospital admission, 7 (17.5%) developed respiratory symptoms during the hospital stay, and 6 (15.0%) had no respiratory symptom. No false positive or false negative tests were found during the first and the second wave.

**Table 1:** Study population.

	Pre-COVID (*N* = 209)	First Wave (*N* = 172)		Transition (*N* = 85)		Second Wave (*N* = 233)	
Outcome	No. (%) of Patients	No. (%) of Patients	Prevalence Ratio(95% CI)	No. (%) of Patients	Prevalence Ratio (95% CI)	No. (%) of Patients	Prevalence Ratio (95% CI)
Sex							
Female	72 (34.5)	70 (40.7)		32 (37.7)		99 (42.5)	
Male	157 (65.5)	102 (59.3)	0.90 (0.77 to 1.06)	53 (62.3)	0.95 (0.78 to 1.15)	134 (57.5)	0.88 (0.76 to 1.02)
Pertinent GE diagnoses	201 (96.2)	75 (43.6)	0.45 (0.38 to 0.54)	79 (92.9)	0.97 (0.91 to 1.03)	199 (85.4)	0.89 (0.84 to 0.94)
Admissions from domicile	124 (59.3)	27 (15.7)	0.26 (0.18 to 0.38)	38 (44.7)	0.75 (0.58 to 0.98)	95 (40.8)	0.69 (0.57 to 0.83)
Mortality	1 (0.5)	7 (4.1)	8.51 (1.06 to 68.5)	3 (3.5)	7.38 (0.78 to 69.9)	13 (5.6)	11.66 (1.54 to 88.4)
Patients undergoing ≥ 1							
endoscopic procedure	98 (46.9)	31 (18.0)	0.38 (0.27 to 0.55)	44 (51.8)	1.10 (0.86 to 1.42)	96 (41.2)	0.88 (0.71 to 1.08)
IR procedure	69 (33.0)	26 (15.1)	0.46 (0.31 to 0.69)	29 (34.1)	1.03 (0.73 to 1.47)	67 (28.8)	0.87 (0.66 to 1.15)

During the first wave, among the 36 physicians that answered the questionnaire, 6 (16.7%) resulted positive to a nasopharyngeal swab and 1 (2.8%) to the serologic tests (Figure 1). No correlation was found between such SARS-CoV-2 testing and the healthcare workers attending the gastroenterological ward activities.

**Figure 2:**
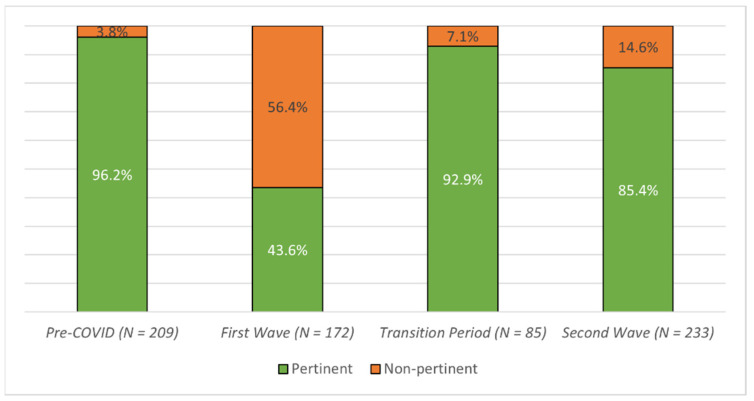
Proportions of pertinent gastroenterological discharge diagnoses.

Compared to the pre-COVID era (96.1%), the gastroenterological pertinence of hospitalized patients decreased both during the first (43.6%) and the second (85.4) COVID-19 wave, while it was similar to the pre-COVID era during the transition period (92.9%) (Figure 2, Table 1). The same trend was observed for admissions from domiciles (Supplementary Figure S1, Table 1). Endoscopic and interventional radiology procedures dropped during the first wave, going back to normal levels during the transition period and the second wave (Supplementary Figure S7, Table 1). The median age at the admission raised during the first and the second wave as well (Supplementary Figure S3, Table 2), while the median CCI raised only during the first wave (Supplementary Figure S5, Table 2). Compared to the pre-COVID era, mortality and the median length of stay increased during all the following periods (Supplementary Figures S2 and S6, Table 1 and Table 2). For the discharge diagnoses of the deceased patients, see Supplementary Table S1.

**Table 2:** Length of stay and Charlson comorbidity index.

	Pre-COVID (*N* = 209)	First Wave (*N* = 172)	Transition (*N* = 85)	Second Wave (*N* = 233)	Total	*p*
Outcome	Median (Range)	Median (Range)	Median (Range)	Median (Range)	Median (Range)	
Age (years)	65.0 (22.5 to 92.2)	73.0 (18.8 to 98.9)	64.0 (17.0 to 88.0)	69.0 (18.5 to 96.0)	68.0 (17.0 to 98.9)	<0.001
Length of stay (days)	4.0 (0.0 to 201.0)	5.5 (0.0 to 40.0)	7.0 (1.0 to 61.0)	6.0 (0.0 to 52.0)	5.0 (0.0 to 201.0)	0.001
Charlson comorbidity index	5.0 (0.0 to 12.0)	6.0 (0.0 to 16.0)	5.0 (0.0 to 11.0)	5.0 (0.0 to 11.0)	5.0 (0.0 to 16.0)	0.02

## 4. Discussion

This report clearly shows a remarkable metamorphosis of a COVID-free gastroenterological ward in the area with the highest European SARS-CoV-2 incidence during the first COVID-19 outbreak. Hospitalized patients were older, with more comorbidities, and they were mostly affected by Internal and Geriatric disorders. Hospitalizations were longer and characterized by higher mortality compared to the pre-COVID era. Coherently, elective admissions and endoscopic or dedicated interventional radiological procedures decreased, reflecting the cancellation of all deferrable procedures [6,7,8,9] and the relocation of most gastroenterological resources (beds, facilities, instrumentations, healthcare personnel) to the prevaricating care needs linked to the pandemic. Another aspect highlighted in our study is the importance and efficacy of regular active surveillance of patients and healthcare personnel with nasopharyngeal swabs and the use of second-level single-use PPE. Indeed, these strategies learned from experience during the first wave, once applied routinely when the second wave began, have led to a significant decrease in the positive cases among patients admitted in our units (in-hospital positive test within day 9) and in the rate of COVID positivity during hospitalization (possible hospital-acquired infection from day 10 to 14, definite hospital-acquired infection from day 15; Supplementary Table S2 and Supplementary Figure S8) along with the decrease in healthcare personnel infection. Notably, when the first SARS-CoV2 wave invested in the metropolitan area of Milan (March 2020), there were no developed isolation protocols nor recommendations on the systematic use of PPE and SARS-CoV2 testing for patients and healthcare personnel with no history of direct contact with confirmed cases regardless of the presence of respiratory symptoms [10]. Consistently, the first measures adopted to face the COVID-19 outbreak in that area (Supplementary methods) were the result of expert consensus based on limited real-life or published evidence and were updated or refined almost day-by-day and with heterogeneity across different hospitals according to the changing availability of human (e.g., intensive care personnel) and instrumental resources (e.g., PPE, respirators, COVID-free facilities). This reflects the scenario of the sudden and unexpected metamorphosis of any hospital protocols that shocked at any level the clinical practice with remarkable impacts on either hospitalized patients’ outcomes, outpatient care continuity, or healthcare personnel daily practice and safety. Moreover, the implementation of preventive measures allowed a satisfactory recovery of elective admissions, endoscopic and interventional radiology procedures during the second wave. This was reflected in the rise of pertinent gastroenterological discharge diagnoses in the transition period and in the second wave compared with the first wave, despite a non-inferior impact of COVID-19 cases on the regional healthcare system [11].

As with all retrospective studies, the present one allows for rapid analysis of the outcomes to find answers for the current scientific needs present in a state of emergency at a global level. One limitation is the possible heterogeneity of data not systematically collected by multiple healthcare professionals. Moreover, considering the unique geographical and temporal setting, no generalization of these results can be made. 

## 5. Conclusions

Overall, our study suggests that active surveillance with repeated SARS-CoV-2 testing and the systematic adoption of second-level single-use PPE when visiting patients are effective measures to control the spread of the virus in a hospital setting. In addition, our experience clearly demonstrates the nonobvious ability to maintain a balance between the need for beds to hospitalize COVID-19 patients and the necessity of continuing practicing medicine during the pandemic to avoid the effects that postponing the activities left behind would have on specific frail populations (e.g., those affected by cancer, cardiovascular, or other chronic conditions). 

## Supplementary Materials

The following are available online at https://www.mdpi.com/article/10.3390/jcm11010109/s1, Figure S1: Modalities of access. Figure S2: Length of stay in the Gastroenterological Unit. Figure S3: Age at the admission. Figure S4: Gender distribution. Figure S5: Charlson comorbidity index. Figure S6: Mortality. Figure S7: Patients attending at least one diagnostic procedure. Figure S8: Days from admission to diagnosis of SARS-CoV-2 infection. Figure S9: Days from the admission in the Gastroenterological Unit to the diagnosis of COVID-19 shown for each case. Table S1: Discharge diagnoses of deceased patients. Table S2: Days from the admission to the Unit to the diagnosis of COVID-19 during the first wave.
