# Supplementary material for: An Unprecedented Challenge: The North Italian Gastroenterologist Response to COVID-19
Source: J Clin Med. 2021 Dec 25;11(1):109. doi: 10.3390/jcm11010109 (PMC8745083; doi:10.3390/jcm11010109)
Supplement: Supplementary file 1 [file jcm-11-00109-s001.zip › jcm-1460772-supplementary.pdf]

Journal of
Clinical Medicine

MDPI

Article

An Unprecedented Challenge: The North Italian Gastroenterologist Response to COVID-19

Gian Eugenio Tontini [1,2,*], Giovanni Aldinio [1,2], Nicoletta Nandi [1,2], Alessandro Rimondi [1,2], Dario Consonni [3], Massimo Iavarone [4], Paolo Cantù [2], Angelo Sangiovanni [4], Pietro Lampertico [2,4] and Maurizio Vecchi [1,2]

[1] Gastroenterology and Endoscopy Unit, Fondazione IRCCS Ca' Granda Ospedale Maggiore Policlinico, Milan, 20100, Italy; giovanni.aldinio@unimi.it (G.A.); nicoletta.nandi@unimi.it (N.N.); alessandro.rimondi@unimi.it (A.R.); maurizio.vecchi@unimi.it (M.V.)
[2] Department of Pathophysiology and Organ Transplantation, University of Milan, Milan, 20100, Italy; paolo.cantu@policlinico.mi.it (P.C.); pietro.lampertico@unimi.it (P.L.)
[3] Epidemiology Unit, Fondazione IRCCS Ca' Granda Ospedale Maggiore Policlinico, Milan, 20100, Italy; dario.consonni@policlinico.mi.it;
[4] Gastroenterology and Hepatology Unit, Fondazione IRCCS Ca' Granda Ospedale Maggiore Policlinico, Milan, 20100, Italy; massimo.iavarone@policlinico.mi.it (M.I.); angelo.sangiovanni@policlinico.mi.it (A.S.)
* Correspondence: gianeugeniotontini@gmail.com

Supplementary file

Supplementary methods

Safety protocols adopted in the gastroenterological ward during the SARS-CoV-2 outbreaks:

- Isolation measures (from March 23, 2020): no entry to non-inpatients; single room for "grey" patients (i.e., those who had only one negative RT-PCR test for SARS-CoV-2 at the admission or with respiratory symptoms while being admitted for less than 5 days); optimization of the healthcare personnel to avoid crowding in the common rooms.
- Active surveillance measures (from March 30, 2020): systematic testing of asymptomatic healthcare personnel and inpatients (before their entrance in the wards, then one and three times per week, respectively).
- Personal protective equipment (PPE): systematic adoption of PPE (i.e., FFP1 mask, gloves, single-use scrub suit) at the ward entrance (from March 13, 2020); systematic adoption of second-level single use PPE (i.e., FFP2 mask plus single-use FFP1 mask, double-gloving, visor, waterproof single-use scrub suit) while visiting each "grey" patient (from March 23, 2020) and the subsequent systematic adoption of the same second-level single use PPE while visiting all the patients (from April 3, 2020).

Supplementary figures

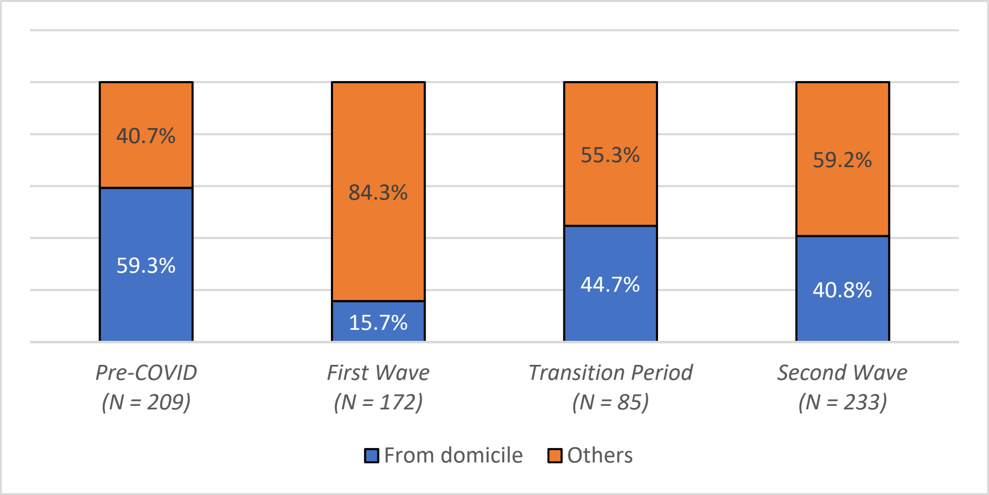

Figure S1. Modalities of access. Respect to the pre-COVID era, for each period (first wave, transition period and second wave), the results are: PR 0.26, 95% CI 0.18 to 0.38; PR 0.75, 95% CI 0.58 to 0.98; PR 0.69, 95% CI 0.57 to 0.83.

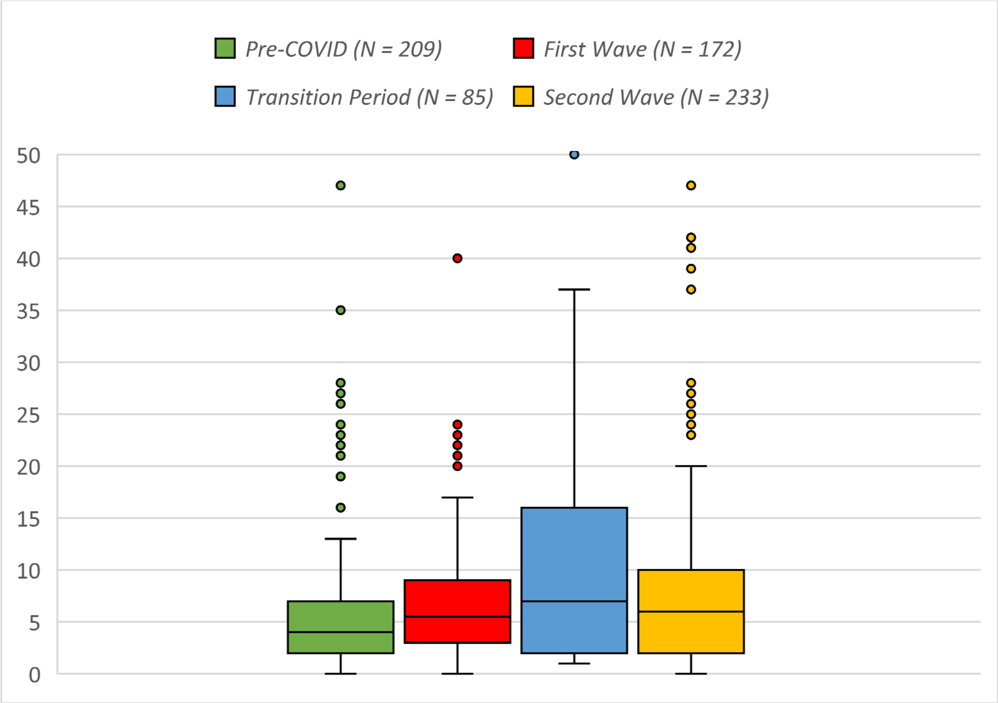

Figure S2. Length of stay in the Gastroenterological Unit. Note that patients >50 days are not represented (out of scale). p = 0.001. The circles are the outliers.

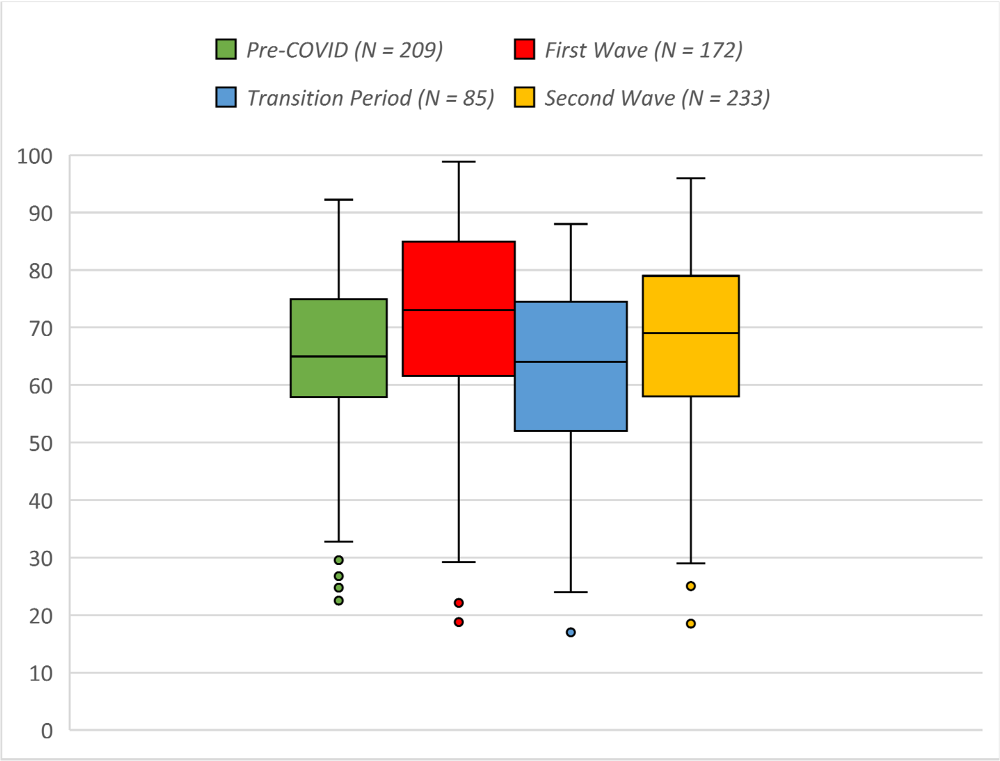

Figure S3. Age at the admission. $p < 0.001$. The circles are the outliers.

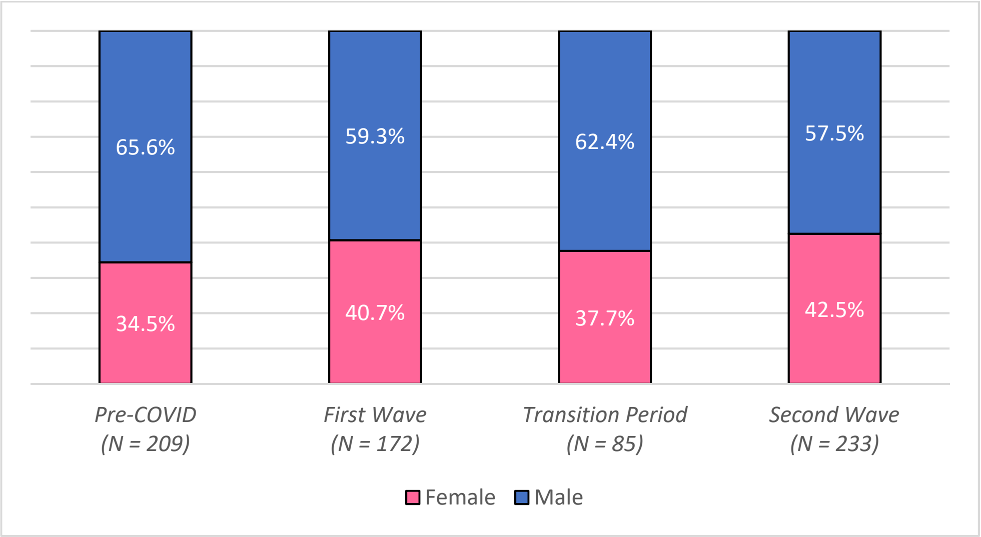

Figure S4. Gender distribution; Respect to the pre-COVID era, for each period (first wave, transition period and second wave), the results are: PR 0.90, 95% CI 0.77 to 1.06; PR 0.95, 95% CI 0.78 to 1.15; PR 0.88, 95% CI 0.76 to 1.02.

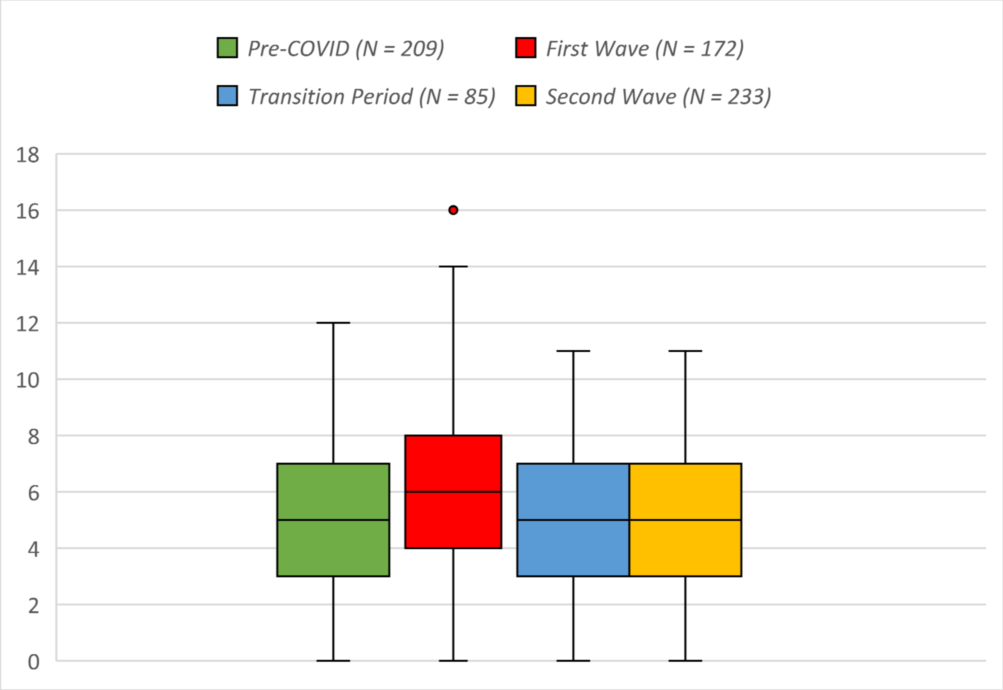

Figure S5. Charlson comorbidity index. $p = 0.02$. The circles are the outliers.

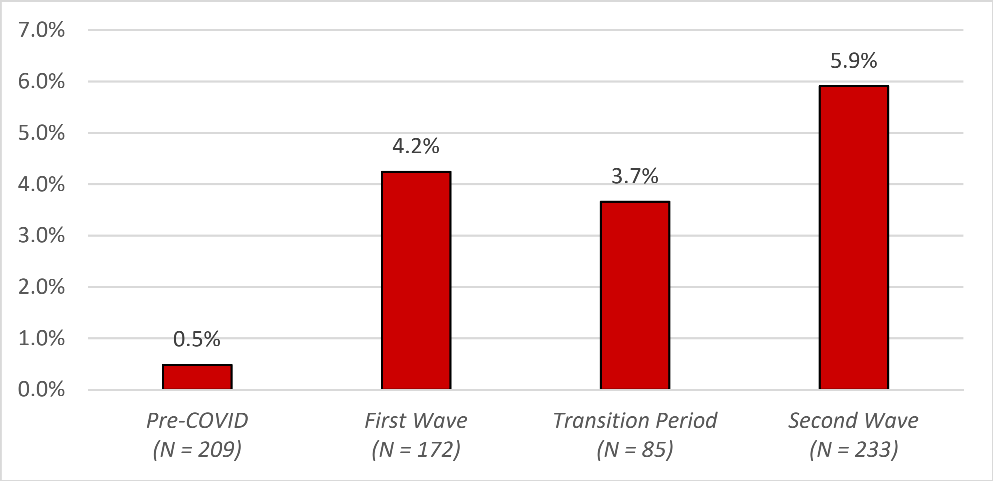

Figure S6. Mortality. Respect to the pre-COVID era, for each period (first wave, transition period and second wave), the results are: PR 8.51, 95% CI 1.06 to 68.5; PR 7.38, 95% CI 0.78 to 69.9; PR 11.66, 95% CI 1.54 to 88.4.

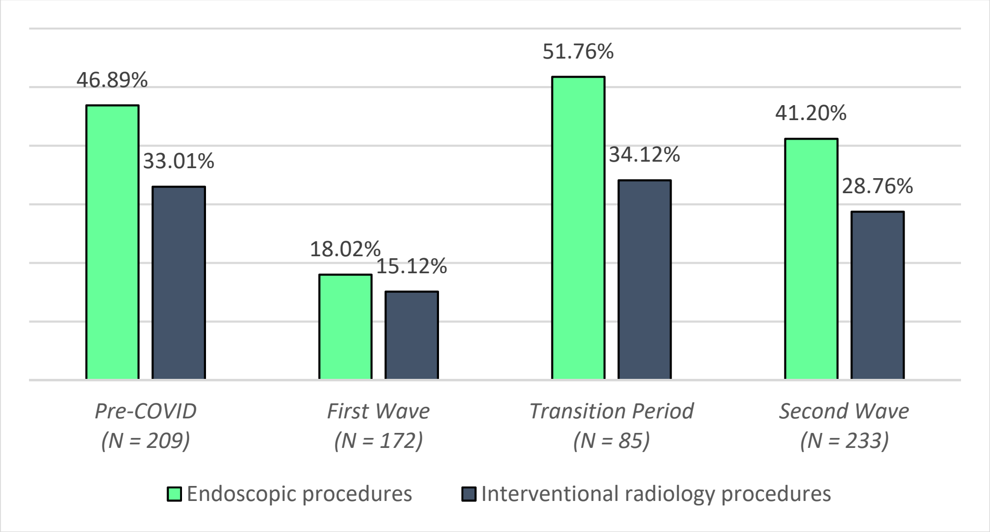

Figure S7. Patients attending at least one diagnostic procedure. Respect to the pre-COVID era, for each period (first wave, transition period and second wave), the results related to the endoscopic procedures are: PR 0.38; 95% CI 0.27 to 0.55; PR 1.10; 95% CI 0.86 to 1.42; PR 0.88; 95% CI 0.71 to 1.08. Respect to the pre-COVID era, for each period (first wave, transition period and second wave), the results the results related to the interventional radiology procedures are: PR 0.46; 95% CI 0.31 to 0.69; PR 1.03; 95% CI 0.73 to 1.47; PR 0.87; 95% CI 0.66 to 1.15.

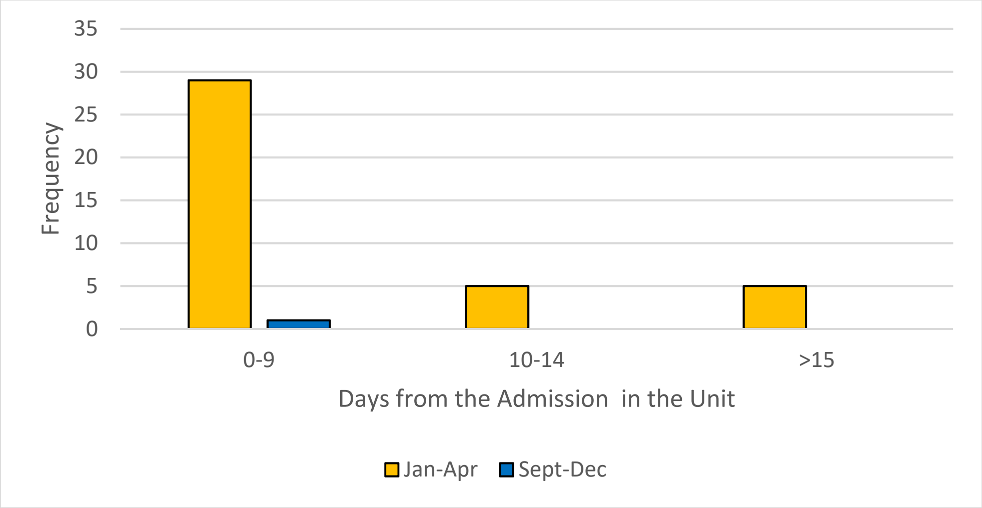

Figure S8. Days from admission to diagnosis of SARS-CoV-2 infection.

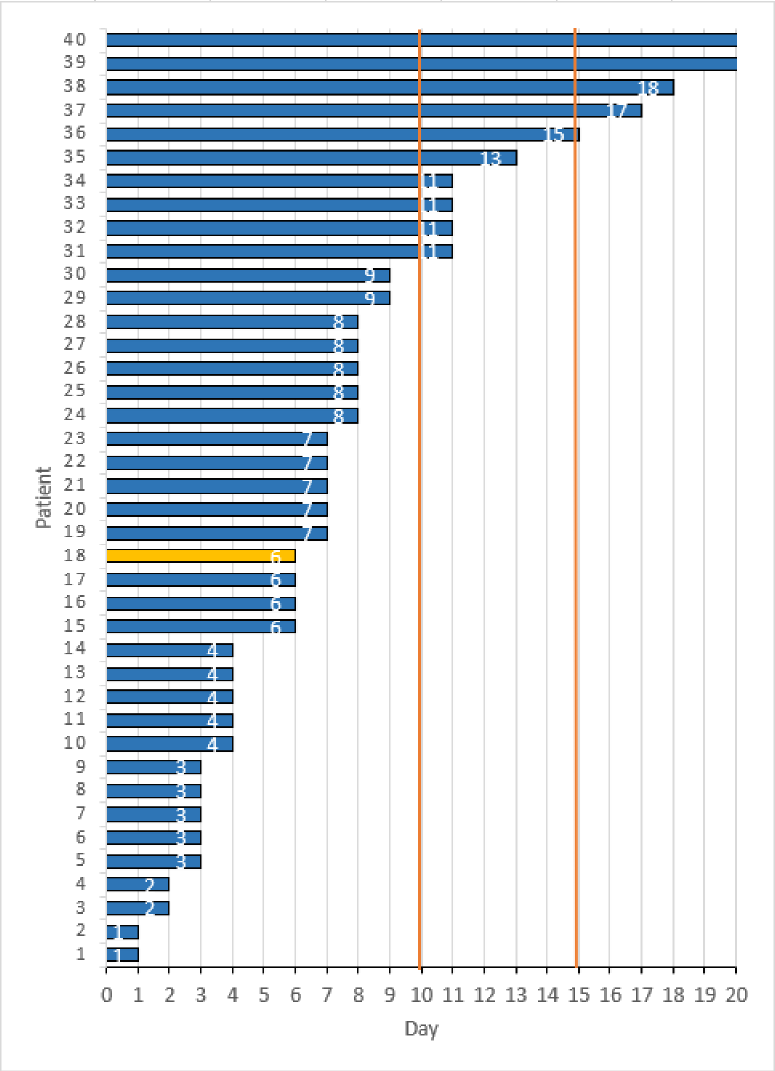

Figure S9. Days from the admission in the Gastroenterological Unit to the diagnosis of COVID-19 shown for each case. The orange vertical lines divide the patients into three categories (0-9, 10-14 and >15 days). In yellow, the only patient resulted positive during the second wave.

Supplementary tables

Table S1. Discharge diagnoses of deceased patients.

Diagnosis at the discharge	No. of deaths
**Pre-COVID era**	
Advanced gallbladder cancer	1
**First wave**	
Congestive heart failure	2
Pneumonia	2
Metastatic hepatocellular carcinoma	2
Peritoneal carcinomatosis of unknown origin	1
**Transition period**	
Sepsis	1
Acute alcoholic hepatitis	1
Sepsis and cholangiocarcinoma	1
**Second wave**	
Refractory celiac disease and lymphoma	1

Hepatocellular carcinoma	1
Cachexia and pancreatic necrosis	1
Renal failure	2
Septic shock	1
Hepatic abscess and chronic cholecystitis	1
Aspiration pneumonia	1
Metastatic lung cancer	1
Hepatocellular carcinoma	1
Pneumonia and metastatic prostate cancer	1
Liver failure	1
Pneumonia	1

Table S2. Days from the admission to the Unit to the diagnosis of COVID-19 during the first wave.

Days	N. of patients	%
0-9	29	74.4%
10-14	5	12,8%
≥15	5	12,8%
